# Discrepant findings of prenatal diagnostics in a case of fetal partial trisomy 21 and fetoplacental mosaicism

**DOI:** 10.1186/s13039-026-00762-7

**Published:** 2026-04-11

**Authors:** T. Dittrich, R. Wenzel, A. Beck, S. Dahlum, R. Siebert, U. Friebe-Hoffmann, S. Bens

**Affiliations:** 1 Institute of Human Genetics, Ulm University and Ulm University Hospital, Ulm, 89081 Germany; 2 Department of Prenatal Medicine and Ultrasound, Ulm University Hospital, Ulm, 89075 Germany; 3 Institute of Pathology, Ulm University Hospital, Ulm, 89081 Germany; 4 Center for Rare Diseases (ZSE), Ulm, Germany; 5 German Center for Childhood and Adolescent Health (DZKJ), Ulm Site, Ulm, Germany

**Keywords:** Down syndrome, Partial trisomy 21, Segmental duplication, Non-invasive prenatal testing (NIPT), Feto-placental mosaicism, Down syndrome critical region (DSCR), Prenatal diagnosis, Genotype-phenotype correlation

## Abstract

**Background:**

Partial trisomy 21 is a rare chromosomal aberration that can provide unique insights into genotype-phenotype correlations in Down syndrome (DS). While non-invasive prenatal testing (NIPT) has become a widely used screening tool for common aneuploidies, its sensitivity is limited in cases of low-level mosaicism or partial duplications. We report a *de novo* partial trisomy 21 encompassing the entire Down syndrome critical region (DSCR) that escaped NIPT.

**Case presentation:**

A 38-year-old first gravida underwent NIPT for evaluation of fetal trisomies 13, 18 or 21 as well as monosomy X, which showed normal results. Ultrasound at 19 + 5 weeks of gestation revealed polyhydramnios and a left-sided fetal hydrothorax, prompting amniocentesis. By karyotyping, a derivative chromosome 21 carrying additional material on the p-arm was identified. PCR-based microsatellite analysis, FISH and array-based studies on amniotic cells characterized the attached material as a *de novo* terminal duplication of 11.8 Mb of chromosome 21q22.12q22.3, fully encompassing the DSCR and without evidence of mosaicism. Comprehensive genetic counselling was provided, and the pregnancy was electively terminated at 24 weeks of gestation. Postmortem examination of the fetus revealed phenotypic features consistent with Down syndrome. In contrast to the non-mosaic duplication observed in amniotic fluid cells, post-termination tissue analysis demonstrated placental mosaicism, comprising predominantly a normal male cell line and a chromosomal aberrant male cell line with partial duplication of 21q22.12q22.3 in 28% of interphase nuclei in short-term cultured chorionic villi. This constellation likely explains the normal NIPT result.

**Conclusion:**

This case illustrates the limitations of NIPT in detecting partial trisomies and low-grade placental mosaicism. It emphasizes the importance of further invasive prenatal investigations when results of ultrasound and NIPT are discrepant and it contributes to the understanding of genotype-phenotype correlations in partial trisomy 21. Clinicians should be aware that partial duplications of 21q22 can produce a full DS phenotype despite unremarkable NIPT results. This case highlights the challenges of technical choices in prenatal diagnostics and emphasizes the need for careful interpretation of screening tests and targeted counselling.

## Background

Trisomy 21 is the most common autosomal aneuploidy in humans and the primary cause of Down syndrome (DS). The phenotype characteristic for DS includes distinctive dysmorphic features, developmental delay, intellectual disability and an increased risk for comorbidities [1]. While most cases result from complete trisomy 21, partial duplications of chromosome 21 are rare and provide valuable insights into genotype-phenotype correlations [2]. In particular, the Down syndrome critical region (DSCR) in 21q22.13 has been identified as a key contributor to the classic DS phenotype [3].

Non-invasive prenatal testing (NIPT) is increasingly used as a screening tool for chromosomal aberrations. It demonstrates high sensitivity and specificity for common numerical aberrations involving chromosomes 13, 18, 21, and sex chromosomes [4]. However, certain clinical scenarios can limit its reliability, potentially leading to discrepancies between NIPT results and invasive diagnostic findings [5]. The cell-free “fetal” DNA analyzed in NIPT originates from the placental trophoblast. Thus, it is more accurately referred to as cell-free placental DNA, providing only an indirect assessment of the fetal karyotype [6]. One major challenge is fetoplacental mosaicism, a condition affecting approximately 0.5% of pregnancies [7]. This constellation can result in discordant findings between placental DNA-based NIPT and the fetal genotype.

Here, we report a rare case of a *de novo* partial trisomy 21 encompassing the entire DSCR, in which low-level placental mosaicism resulted in a normal NIPT. This case highlights the limitations of NIPT. Moreover, it underscores the importance of invasive prenatal testing when sonographic abnormalities still raise suspicion for chromosomal abnormalities despite negative NIPT results.

## Case presentation

A 38-year-old woman presented at 13 + 5 weeks of gestation of her first pregnancy at our prenatal center for first-trimester screening. The patient had no medical history aside from uterine myomatosis and endometriosis. Her partner was healthy. Prior investigations had revealed fetal nuchal translucency within the upper normal range and normal results for non-invasive prenatal testing (NIPT). The latter test was evaluated for aneuploidies involving chromosomes 13, 18, 21, and the sex chromosomes X and Y.

At first-trimester screening, the fetus showed normal fetal development with nuchal translucency within the normal range. The calculated adjusted risk for trisomies 13, 18, and 21 was 1:3027, 1:6387, and 1:335, respectively, based on maternal background risk, ultrasound markers, and serum biochemistry (free β-hCG and PAPP-A). Incorporation of biochemical markers (free β-hCG 59,900 IU/L (1,9122 MoM (multiple of the median)), PAPP-A 2,460 IU/L (0,3954 MoM)) into the initial calculation increased the estimated risk for trisomy 21 to 1:17. However, after adding additional ultrasound markers (i.e. measurements of nasal bone, tricuspid and ductus venosus flow) to the risk calculation, the risk reduced to 1:335.

Amniocentesis was discussed with the couple, but because of normal NIPT result they opted for regular second trimester screening by a prenatal diagnostics specialist. At the follow-up ultrasound at 19 + 5 weeks of gestation, polyhydramnios was observed. The fetus presented with a left-sided hydrothorax. Fetal echocardiography was unremarkable, and overall fetal development was appropriate for gestational age. Due to the upper listed results of the first trimester screening, amniocentesis was again debated with the parents and subsequently performed and genetic analyses initiated.

**Fig. 1:**
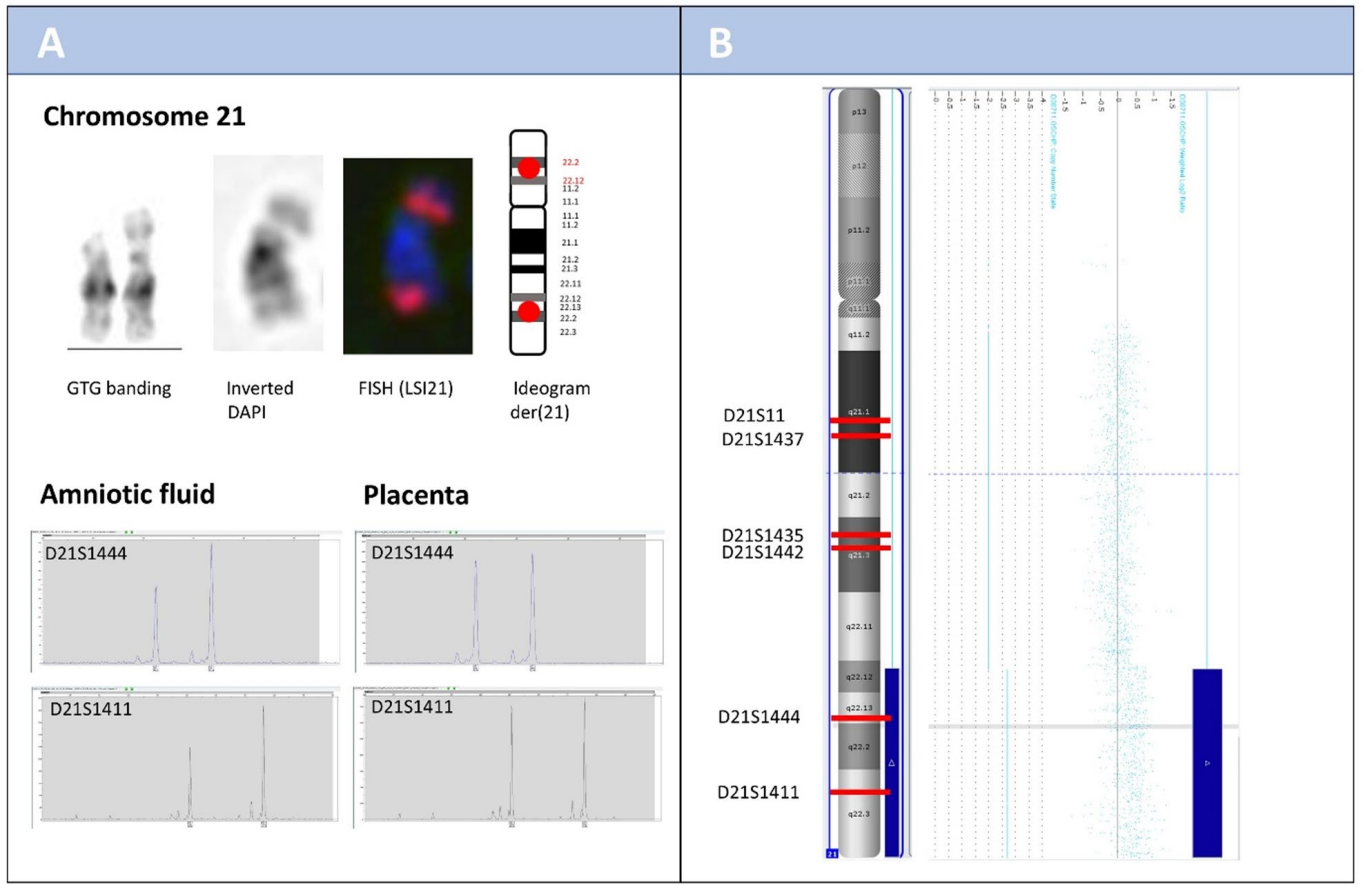
**A**: Partial karyotype of the fetus showing a derivative chromosome 21 with a duplicated segment (21q22.12–q22.3) attached to the p-arm at 21p, identified by GTG banding. Fluorescence in situ hybridization (FISH) signal pattern on the derivative chromosome 21 of amniotic fluid cells using the LSI21 probe (D21S342/D21S341/D21S259; Abbott/Vysis, Wiesbaden) revealed two fluorescence signals on the derivative chromosome 21: one at the expected 21q locus and an additional signal at 21p, indicating duplication of 21q22 and translocation of the duplicated segment to 21p. An ideogram of the derivative chromosome 21 illustrates the LSI21 probe localization. Quantitative fluorescence PCR (QF-PCR) results from amniotic fluid (left) and chorionic villi (right) are shown in the lower part of A. In amniotic fluid, STR markers D21S1444 and D21S1411 show a 2:1 allele ratio, consistent with partial trisomy 21 (i.e. three copies). In chorionic villi, the same markers exhibit slightly different peak heights (ratio of peak areas 0.9366 (D21S1411) and 0.922 (D21S1442)). These ratios lie still in the limits of the normal range (0,75 − 1,44 according to user guidelines), but ratio differences to those markers outside the duplicated region (peak area ratios of 1.0066 (D21S1442), 0.9656 (D21S1435), 1.0132 (D21S1437), and 1.0044 (D21S11), data not shown) may still reflect the confirmed mosaic constellation. **B**: OncoScan array confirming an 11.8 Mb duplication of 21q22.12–q22.3 (vertical blue bar), corresponding to the ISCN 2024 karyotype: arr[GRCh37] 21q22.12q22.3(36,285,911_48,097,610)x3. The array plot shows Log_2_ ratio values across chromosome 21, with elevated values (~ 0.5) consistent with three copies of the duplicated segment. An ideogram of chromosome 21 illustrates the duplicated region (vertical blue bar), and the positions of the relevant QF-PCR markers are indicated as red lines.

PCR-based microsatellite analysis (Devyser Compact V3, Dvysr^®^, Arsta, Sweden) performed on uncultured amniotic cells revealed for two out of five informative markers a signal pattern suggestive of a partial gain of chromosomal material on chromosome 21 (Fig. 1A). These two markers are located in region 21q22.13 (D21S1444) and 21q22.3 (D21S1411) (Fig. 1A). The remaining three informative markers for chromosome 21 showed normal disomic signal patterns. Since both markers with altered signal pattern map consecutively to the terminal region of the long arm (q-arm) of chromosome 21 (Fig. 1B), the findings were suggestive for a terminal partial duplication in this region.

Karyotyping after GTG banding on in-flask cultured amniotic fluid cells revealed in 8 metaphases of one culture an abnormal male karyotype with a derivative chromosome 21 harboring additional chromosomal material of unknown material on its short arm (Fig. 1A). Since PCR-based microsatellite analysis indicated a partial terminal gain in 21q, further FISH studies were performed. We applied probes specific for chromosomes 13 (control) and 21 hybridizing to regions 13q14 and 21q22.13q22.2, respectively (LSI 13 (RB1)/21 (D21S342/D21S341/D21S259), Abbott/Vysis (AneuVysion kit), Wiesbaden). A total of 100 interphases and 15 metaphases of cultured amniotic cells were analyzed. Interphase nuclei showed three signals for the chromosome 21-specific probe in 92% of cells (92/100), which was interpreted as consistent chromosomal aberration. In metaphase spreads, two signals were observed on the derivative chromosome 21: one in its expected location and one ectopically located within the additional material on 21p (Fig. 1A). A normal signal constellation was present on the structurally intact chromosome 21. The karyotype was initially described in accordance with ISCN 2024 as 46,XY, der(21)t(21;21)(p1?;q22).ish der(21)t(21;21)(p1?;q22)(D21S342/D21S341/D21S259+;D21S342/D21S341/D21S259+).

Thus, a duplication of chromosome 21q was confirmed, encompassing at least the chromosomal region 21q22.13 to 21q22.2. To further characterize the size of the duplication, molecular karyotyping (OncoScan array analysis (Thermo Fisher Scientific Inc., Life Technologies GmbH, Darmstadt, Germany)) was performed. Evaluation of the array analysis was limited to copy number gains and losses exceeding 2 Mb in size. The molecular karyotyping revealed a terminal gain of 11.8 Mb involving chromosomal region 21q22.12q22.3 (Fig. 1B). This corresponds to the following ISCN 2024 karyotype: arr[GRCh37] 21q22.12q22.3(36,285,911_48,097,610)x3. Within the gained region, 189 genes are located. Of these, 79 are listed in OMIM as being associated with human disease. Notably, the Down syndrome critical region (DSCR) 21q22.13 is fully encompassed within the duplicated segment [3].

To investigate whether the fetal chromosomal aberration represented a *de novo* event, chromosomal analysis and FISH diagnostics of phytohaemagglutinin (PHA)-stimulated lymphocyte cultures were performed in both parents. Conventional karyotyping revealed a normal female karyotype in the mother and a normal male karyotype in the father. FISH analysis was conducted using probes targeting the *RUNX1* locus (LSI RUNX1/RUNX1T1, hybridizing to regions 21q22/8q21.3, Abbott/Vysis), which is located within the genomic region duplicated in the fetus, as well as with probes for chromosomes 13 and 21 (LSI 13/21 (AneuVysion kit, Wiesbaden, see above). In both hybridizations, signal number and localization were analyzed in 100 inter- and 15 metaphases, respectively. For all probes used results were consistently unremarkable in both parents, indicating most likely a *de novo* chromosomal structural rearrangement in the fetus.

Comprehensive genetic counselling was conducted. All identified genetic findings, as well as the anticipated phenotype of the child — most likely consistent with the full clinical presentation of Down syndrome — were discussed with the couple. Taking all discussed aspects into consideration, the couple ultimately opted for termination of pregnancy. This was performed at 24 weeks of gestation in accordance with the regulations of the German law.

Post-termination analysis of various fetal tissues (placenta, Achilles tendon, and spleen) was performed using a PCR-based test (Devyser Compact V3 Dvysr^®^, Arsta, Sweden). In the Achilles tendon and spleen samples, the signal pattern paralleled that in the amniotic fluid sample. Two out of five informative markers (D21S1444 and D21S1411) on chromosome 21 indicated a partial trisomy 21 involving regions 21q22.13 and 21q22.3. No evidence for mosaicism was noted. Contrastingly, the same analysis of uncultured placental tissue revealed overall normal results. However, based on peak height evaluation, the results were suspicious for a low-grade mosaicism comprising a minor cell line with partial trisomy 21 alongside a predominant normal male cell line. Nevertheless, sensitivity was insufficient for an unambiguous diagnosis of mosaicism (Fig. 1A). FISH analysis, using LSI 13/21 by Abbott/Vysis (AneuVysion kit), was additionally performed on 18 h-cultured chorionic villi. The analysis of 100 interphase cells of two separate cultures confirmed a mosaic pattern consisting of an aberrant cell line with partial trisomy 21 (28% of the analyzed cells with three copies for the region the LSI 21 probe hybridizes to), alongside a cell line with a normal disomic number of chromosome 21 copies. To exclude maternal cell contamination as a potential origin of the normal cell line, additional probes targeting the X and Y chromosomes (DXZ1/DYZ1, Abbott/Vysis, hybridizing to regions Xp11.1q11.1 and Yq12, respectively) were employed. Analyzed cells consistently exhibited a male signal pattern, thus, excluding maternal contamination and confirming placental mosaicism. Full karyotyping of chorionic villi and fetal Achilles tendon was not successful due to bacterial infection of the cultures and failed growth, respectively.

Post-termination histopathological examination of the placenta revealed a weight between the 10th and 50th percentile, with chorionic villi demonstrating largely appropriate maturational development. At autopsy, the crown-rump length of the fetus was 20.5 cm (reference range at 24 weeks of gestation: 19.7–22.9 cm), and the fetal weight was 560 g (reference range at 24 weeks of gestation: 464–694 g [8]). Internal organs were normally developed. A single transverse palmar crease was present on the left hand. Evaluation of the remaining extremities was limited due to advanced autodigestion with epidermal sloughing. No cardiac malformations were identified. The skeletal system was unremarkable. The liver, kidneys, and adrenal glands were below the expected weight for gestational age. The thymus appeared small on gross inspection but weighed at the lower limit of the normal range. This is in line with the observation that the majority of Down syndrome fetuses have smaller thymuses than controls [9].

In summary, the genetic analyses demonstrated a *de novo* partial trisomy 21 involving the region 21q22.12–q22.3, which entirely includes the DSCR at 21q22.13. This duplicated segment was attached to the short arm of the derivative chromosome 21. Interestingly, the analysis of the placenta showed a mosaic constellation of the detected aberrant cell line in 28% of analyzed cells in short-term-cultured chorionic villi next to a normal male cell line. This could explain the discrepant findings between NIPT and amniocentesis with regard to the diagnosis of fetal Down syndrome and is yet another example for fetoplacental discrepancies in prenatal diagnostic care. Of note, NIPT data was generated by whole-genome sequencing and evaluation was subsequently restricted to fetal aneuploidies of chromosomes 13, 18, 21 and X. A retrospective follow-up with the NIPT provider confirmed that low-grade placental mosaicism would have resulted in a negative NIPT-result, even if partial copy number variations (pCNVs) had been assessed.

## Discussion

In the present case, following a normal non-invasive prenatal test (NIPT), amniocentesis revealed a partial trisomy 21 of 11.8 Mb involving a duplication of the chromosomal region 21q22.12-q22.3. Placental analysis showed a low-level mosaicism of this aberrant cell line alongside a normal male cell line.

The mechanism by which the derivative chromosome might have formed is unknown. However, based on the findings from Bonaglia et al. in 2018 it is most tempting to pursue the hypothesis that the *de novo* unbalanced translocation and mosaic constellation are the result of a simple translocation derived from a complex background. As first event, one might expect meiotic non-disjunction leading to trisomy 21. This might have been followed by postzygotic chromothripsis of the supernumerary chromosome, with preservation of its distal part during trisomy rescue. The remaining distal part was subsequently rescued by translocation to a chromosome 21 that acted as the acceptor chromosome by losing its satellites as seen in the formation of Robertsonian translocations. While we did not perform any molecular investigations in our case, this represents the most common mechanism for *de novo* unbalanced translocations in mosaic states, reported as a class A mechanism by Bonaglia MC et al. in 2018 [10]. If so, one could speculate that a third cell line with free trisomy 21 might have existed in the present case, which we did not detect in our analysis. An additional aspect that could support the aforementioned hypothesis is the advanced maternal age (38 years) in the present case.

Beyond technical challenges that can result in a false NIPT outcome, there are biological factors that can prevent NIPT from accurately reflecting the true fetal genotype. In order to understand the differing results from the prenatal diagnostic work-up, it is important to understand which cell compartment is studied in each analysis. While cfDNA in NIPT and cells in short-term cultured chorionic villi predominantly represent the cytotrophoblast, long-term cultured villi reflect the mesenchymal core, whereas amniotic fluid cells are of fetal origin [11]. Therefore, results from interphase FISH on short-term cultured villi and NIPT likely represent the same cell compartment. According to the current literature, low-level mosaicisms (< 30%) are expected to be missed by NIPT [11]. Our interphase FISH results, showing a fraction of 28% for the aberrant cell line in short-term cultured villi, fit well with this general observation. Van Opstal et al. showed that 2% of fetal trisomy 21 cases may be missed by NIPT due to a normal or low-level mosaic karyotype in the cytotrophoblast [11].

Feto-placental mosaicism is a well-recognized biological cause of discordant NIPT findings. The proportion and distribution of the abnormal cell line within placental tissue determines whether the aberration can be detected in cell-free DNA. In the present case, post-termination testing by FISH confirmed low-grade mosaicism for partial trisomy 21 in placental tissue. Initially, results of all markers analyzed by QF-PCR on DNA of uncultured chorionic villi fell within the normal range for 1:1 ratio. However, Donaghue et al. demonstrated that mosaicism can be reliably detected by QF-PCR when the proportion of abnormal cells exceeds approximately 15% [12]. This indicates that the mosaicism in our case likely fell below this threshold in the native placenta sample. The 28% abnormal cells detected in short-term cultured chorionic villous cells by FISH likely reflect selective growth of the aberrant cell population.

With regard to mosaic trisomy 21 Feresin et al. reported two cases with discordant NIPT and invasive test results. In the first case, both placenta and fetus exhibited mosaic trisomy 21, whereas in the second case, the fetus had full trisomy 21 while the placenta showed mosaic trisomy 21 in approximately 50–60% of cells [13].

In addition to false-negative results, most commonly explained by low fetal fraction or mosaicism as discussed above, false-positive NIPT results also require attention. Since the majority of cfDNA analyzed in NIPT is of maternal origin, false positive results may arise from maternal conditions, including maternal tumors [14]. In this context, it is important to emphasize that NIPT results in such constellations may be analytically correct but still discordant with the fetal genome. Regarding the present case, it is important to note that the patient had uterine myomatosis, a condition known to potentially influence NIPT results. Approximately 40–50% of uterine fibroids have detectable cytogenetic rearrangements [15]. Karyotypically abnormal uterine fibroids have been reported as a source of “false positive” NIPT results that do not reflect the fetal genome [15]. Consequently, it is conceivable that karyotypically normal fibroids may also contribute to false-negative results and might have affected the NIPT outcome observed here.

In the present case, the discrepancy between NIPT and invasive testing with regard to the clinical diagnosis of fetal Down syndrome can be explained by the combination of low-grade placental mosaicism and the presence of a partial, rather than complete, trisomy 21. Given that the duplication included only parts of chromosome 21 and that there was low-level mosaicism in the placenta, NIPT and the evaluation strategy were not sensitive enough to detect this aberration. This limitation is supported by data from genome-wide NIPT studies. A study by Zhen et al. evaluated the sensitivity of genome-wide NIPT designed to detect common aneuploidies, rare autosomal aneuploidies, and partial copy number variations (pCNVs). Detection rates were 97% for whole-chromosome aneuploidies, 63.6% for pCNVs larger than 5 Mb, and 70.6% for fetal mosaicism [16]. These findings indicate that pCNV detection sensitivity remains lower compared to whole-chromosome aneuploidies.

When counselling pregnant women with a negative NIPT result, it is important to discuss the residual risk of a fetal chromosomal aberration. The magnitude of this residual risk depends on several factors, including the design and resolution of the NIPT assay, maternal age, and the presence or absence of abnormalities in prenatal ultrasound. In fetuses with a negative NIPT result and no sonographic abnormalities, the residual risk has been reported to range between 0.14% and 1.07% [17, 18]. This risk increases in the presence of abnormal prenatal ultrasound findings substantially to a range from approximately 2.0% to 8,1% (genome-wide NIPT, resolution 10–20 Mb) or 13.3% (targeted NIPT regarding autosomal aneuploidies 13, 18 and 21) [17, 19]. The variability in reported risk estimates is primarily attributable to differences in study design, inclusion criteria, and the genomic resolution of the NIPT used [17].

Beyond the detection challenges, the partial trisomy also provides insight into genotype-phenotype correlations, particularly regarding the Down syndrome critical region (DSCR). The DS phenotype is characterized by a distinctive pattern of dysmorphic features, developmental delay, intellectual disability and associated comorbidities. While complete trisomy 21 accounts for the classic presentation, partial trisomies provide a valuable model for exploring genotype–phenotype correlations. Genomic mapping of such cases has enabled the identification of chromosomal segments linked to specific traits, leading to the concept of the Down syndrome critical region (DSCR). This framework underpins current discussions on the contribution of individual genes and loci to the Down syndrome phenotype [3, 20, 21].

Delabar et al. first postulated the existence of the DSCR responsible for the characteristic phenotype, mapping 24 clinical features to six minimal regions within 21q22.2 and 21q22.3 [20]. Korbel et al. later analyzed 30 patients with rare segmental trisomies of chromosome 21 and identified several regions associated with features such as congenital heart defects, leukemia, Alzheimer’s disease, and intellectual disability, thereby refuting the notion of a single locus accounting for all phenotypic traits [21]. Pelleri et al. further compared two cases of partial duplications – one including the DSCR leading to DS phenotype and one excluding it without clinical evidence of DS phenotype – demonstrating that duplication of this region is crucial for the classical DS phenotype [3].

In the present case, the duplicated segment (21q22.12-q22.3) entirely encompasses the DSCR, leading to the assumption of classical DS phenotype to occur in the fetus. Unfortunately, phenotype correlation is in our case limited due to advanced autodigestion and lack of data on postnatal development. Thus, facial dysmorphism could not be assessed accurately. Additionally, typical characteristics, i.e. intellectual development or many comorbidities, cannot be evaluated in a prenatal setting. Major malformations were absent; however, this would not exclude full Down syndrome. A single transverse palmar crease was noted, a dysmorphic feature previously associated with duplications involving 21q22.13-q22.3 [22]. A comparable case has been reported in the literature: the patient described carried a 10.9 Mb duplication in the same chromosomal region and exhibited typical DS features, including abnormal palmar creases, facial dysmorphism, and intellectual disability [23]. This further supports the notion that duplication of 21q22.12-q22.3 alone can be sufficient to produce the characteristic Down syndrome phenotype.

Taken together, this case underscores the limitations of NIPT in similar scenarios and highlights the value of integrating genetic findings from different diagnostic approaches with phenotypic assessment.

## Conclusion

In conclusion, our case highlights the diagnostic challenges posed by the coexistence of placental mosaicism and partial trisomy 21. The present case emphasizes that structural aberrations can also occur in mosaic form and that these constellations may remain undetected by NIPT, even if the structural aberration exceeds the resolution limit of the method. The elucidation of seemingly discordant constellations by performing invasive placental examination when NIPT and fetal results differ, helps clinicians and patients to understand that such findings do not necessarily indicate a “false-negative” NIPT result. They may instead result from an abnormal cell fraction below the detection limit or from an inappropriate NIPT design for a given aberration, i.e. if the design does not target partial chromosomal imbalances due to structural aberrations. Thus, there is a continued need for invasive testing, especially in discordant scenarios. Partial trisomies furthermore emphasize the clinical relevance of correlating genomic findings with phenotype, as they provide a unique opportunity to refine genotype-phenotype correlations in Down syndrome and remain an important consideration in the evaluation of atypical presentations. Thus, reporting rare cases like this one is essential to expand the collective knowledge base and improve counselling in complex prenatal diagnoses.

## Data Availability

The datasets generated and/or analyzed during the current study are in main parts included into the paper. All other information not publicly available due to risk of compromising individual privacy is available from the corresponding author on reasonable request.
